# Swallow Syncope as a Late Complication of Sleeve Gastrectomy

**DOI:** 10.7759/cureus.81434

**Published:** 2025-03-29

**Authors:** Fred Rudensky, Sana N Khan, Prasad Chalasani

**Affiliations:** 1 Department of Internal Medicine, HCA Florida Lawnwood Hospital, Fort Pierce, USA; 2 Department of Internal Medicine, HCA Healthcare Medical City Arlington, Arlington, USA; 3 Department of Cardiology, HCA Florida Lawnwood Hospital, Fort Pierce, USA; 4 Department of Cardiology, Florida State University College of Medicine, Tallahassee, USA

**Keywords:** bariatric surgery complications, cardiac pacemaker, deglutition syncope, dual-chamber pacemaker, hiatal hernias, intermittent sinus pause, parasympathetic nervous system, situational syncope, swallow syncope, vertical sleeve gastrectomy

## Abstract

Swallow syncope, also known as swallow-induced syncope, or deglutition syncope, is a type of situational reflex syncope associated with swallowing. It is believed to be due to exaggerated vagal parasympathetic stimulation leading to inhibition of heart rate during swallowing. Swallow syncope has been documented in cases of gastroesophageal structural pathologies, such as achalasia and esophageal stricture, and has even been shown to resolve following surgical correction. To date, there are 118 reported cases of swallow syncope published in medical literature, which includes 14 reports of swallow syncope with associated hiatal hernia, and only one of which reports swallow syncope following sleeve gastrectomy. We present a case of swallow syncope as a late complication of laparoscopic sleeve gastrectomy associated with gradually worsening hiatal hernia.

Our patient is a 54-year-old female who presented with a chief complaint of acute-onset episodic presyncope and syncope that occurs when swallowing food or liquids. Pertinent past medical history includes morbid obesity, obstructive sleep apnea, and a remote history of neurocardiogenic syncope during childhood. Approximately six weeks prior to presentation, the patient underwent laparoscopic sleeve gastrectomy complicated by surgical infection which had since resolved. Asystolic pauses lasting three to four seconds, occurring exclusively during deglutition, and sinus bradycardia with heart rates as low as 20 beats per minute were noted on telemetry monitoring over the course of her hospital admission. Following evaluation by electrophysiology consult, shared decision was made to proceed with dual-chamber pacemaker placement. During a follow-up visit seven months post pacemaker placement, the patient reported no pre-syncopal or syncopal episodes. At that time, a review of the patient's multiple prior imaging studies incidentally revealed a hiatal hernia, which first appeared on a CT image taken eight days after her bariatric procedure. Review of the most recent imaging studies available at that time showed a progressive increase in size of the herniation since its initial appearance.

Our case of swallow syncope, a rare disorder in itself, is made even more anomalous by the underlying etiology of iatrogenic hiatal hernia following sleeve gastrectomy, the patient's family history of swallow syncope secondary to Roux-en-Y procedure, and the decision to treat with dual-chamber pacing. The patient's history of surgical infection also raises the question of whether post-infectious changes are wholly, or partly, responsible for her symptoms. Guideline treatment options such as lifestyle modification and medical management were not attempted due to concern for possible treatment failure and the need for definitive and immediate resolution of the patient's symptoms since quality of life was significantly impacted. Correction of underlying pathology was not considered prior to pacemaker placement due to failure to identify the hiatal hernia as a potential inciting factor in time. Our case highlights the variety of possible etiologies leading to situational syncope, the possibility of a hereditary component, and successful resolution of symptoms following the unconventional decision to treat with pacemaker placement.

## Introduction

Swallow syncope, also known as swallow-induced syncope, or deglutition syncope, is a type of situational reflex syncope associated with swallowing [1]. There have been a total of 117 cases of swallow syncope reported in the literature [2]. It is believed to be due to exaggerated parasympathetic vagal nerve signaling to the sinoatrial and atrioventricular nodes, leading to the suppression of heart rate during deglutition [2,3]. Lower esophageal mechanoreceptors are stimulated by the passing of food or liquid bolus causing afferent signaling to the adjacent motor nucleus, leading to efferent vagal nerve parasympathetic signaling causing suppression of heart rate and contractility [3]. Swallow syncope has been documented in cases of gastroesophageal structural pathologies, such as achalasia and esophageal stricture, and has even been shown to resolve following surgical correction of the underlying gastroesophageal issues [2]. To date, there are at least 19 reports of swallow syncope with associated hiatal hernia [2,4], and at least one report of swallow syncope following vertical sleeve gastrectomy [5]. We present a case of swallow syncope as a late complication of laparoscopic sleeve gastrectomy with subsequent gradually increasing hiatal hernia confirmed on CT imaging.

## Case presentation

Our patient is a 54-year-old female who presented with a chief complaint of episodic presyncope and syncope that occurs when swallowing food or liquids. Pertinent past medical history included laparoscopic sleeve gastrectomy complicated by surgical infection six weeks prior to presentation, morbid obesity, obstructive sleep apnea, and a remote history of neurocardiogenic syncope during childhood. The patient described the childhood neurocardiogenic syncope as one or two episodes of syncope due to emotional distress which did not require intervention, but could not elaborate further. The patient described her presenting symptoms as sudden fogginess, dizziness, and loss of consciousness lasting approximately 30-45 seconds. She stated the episodes were not caused by any specific foods and began approximately 10 seconds after swallowing. She also stated that her smart watch alerted her to bradycardia on multiple occasions but could not recall the lowest recorded rate. She denied any other symptoms associated with the syncopal episodes including chest pain, palpitations, abdominal pain, or diaphoresis. She denied tobacco, alcohol, or drug use. Of note, the patient reported her twin sister had undergone Roux-en-Y gastric bypass procedure and subsequently developed swallow syncope. Swallow syncope was formally diagnosed, although it is not known if she had any anatomical structural abnormalities such as hiatal hernia.

Review of home prescriptions did not show any medications which increased risk of bradyarrhythmia. Prior medical records revealed a recent transthoracic echocardiogram showing normal ventricular function and an ejection fraction of approximately 60% without evidence of wall motion abnormalities or structural defects. Electrocardiogram from date of admission showed sinus bradycardia with a heart rate of 55 beats per minute with no other abnormal findings and no significant changes from prior studies. Doppler ultrasound of the carotid arteries showed minimal plaque formation with patent flow bilaterally. Review of EKGs and vital signs from prior studies did reveal occasional sinus bradycardia with heart rates ranging from 50-60 beats per minute. Although attempts at reproducing patient’s syncopal episodes under visual observation were not made due to safety concerns, nursing staff did report witnessing multiple episodes. Asystolic pauses lasting three to four seconds and sinus bradycardia with heart rates as low as 20 beats per minute were recorded on telemetry monitoring during the patient’s hospital stay. All of the asystolic pauses and episodes of bradycardia below 50 beats per minute occurred exclusively during ingestion of food or liquid. The patient’s respiratory rate and blood pressure remained stable and within normal limits during these episodes.

**Figure 1:**
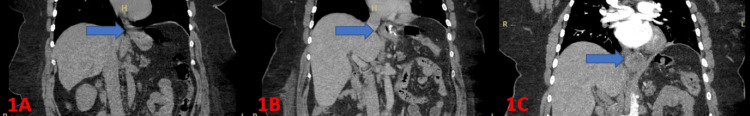
CT imaging showing progressive hiatal hernia A: CT of the abdomen and pelvis showing absence of a hiatal hernia (blue arrow) 13 months prior to the patient's laporascopic sleeve gastrectomy. B: CT of the abdomen and pelvis showing presence of a hiatal hernia (blue arrow) eight days after the patient's laparoscopic sleeve gastrectomy C: CT angiogram of the chest showing progressive enlargement of a hiatal hernia (blue arrow) two months after the patient's laparoscopic sleeve gastrectomy

CT Angiogram of the chest did not reveal any acute cardiopulmonary process, but did show a hiatal hernia adjacent to the left atrium (Figure 1). No further imaging studies were deemed necessary at that time, although later review of CT imaging from the patient’s prior admissions revealed that the hiatal hernia had gradually increased in size since the first appearing on CT imaging from five weeks prior. The hiatal hernia was not present on imaging prior to her bariatric procedure (Figure 1). No pertinent laboratory abnormalities were noted. The patient was found to have markedly low heart rate variability, as well as minimal change to heart rate when transitioning from recumbent position to standing and ambulating, indicating possible autonomic dysfunction although formal testing with tilt table test or deep breathing test was not conducted. Following evaluation by electrophysiology consult, a shared decision was made to proceed with dual chamber pacemaker placement. 

As per the anesthesia report from the date of pacemaker implantation, the patient’s heart rate was 58 immediately prior to administration of midazolam, propofol, and succinylcholine. Following anesthesia induction and intubation, the patient’s sinus bradycardia gradually worsened although rhythm remained sinus. Once the atrial lead was advanced to the chosen area of implantation, the patient’s rhythm became junctional with a rate of 34 beats per minute requiring temporary pacing set to a rate of 60 beats per minute. The pacer was implanted and a minimum rate of 50 beats per minute was set. No complications were noted following the procedure and the patient quickly recovered. She was discharged the following day and instructed to follow up with electrophysiology consult and her primary care provider.

Review of EKG studies from emergency department visits and hospital admissions for unrelated complaints in the months following pacemaker implantation showed alternating sinus bradycardia and normal sinus rhythm, although heart rate did not decrease below 57 beats per minute. The patient reports still experiencing intermittent bradycardia but states she has not experienced any presyncopal or syncopal episodes seven months post pacemaker placement, although swallow-induced bradycardia was not formally assessed after implantation.

## Discussion

In a literature review of 101 cases of swallow syncope, atrioventricular blocks were noted in 34.7% of patients while sinus dysfunction was noted in 33.7% [2]. Of the 101 cases, 32.7% of patients presented with associated gastrointestinal comorbidities [2]. Although there is only one report of swallow syncope associated with bariatric procedure prior to the current case [5], sinus bradycardia is strongly associated with bariatric surgery, with one study estimating approximately 15% of patients developing vasovagal syncope following bariatric procedure [6], and one study estimating prevalence of bradyarrythmia following bariatric procedure to be approximately 18% [7]. 

While not entirely understood, the central cause of swallow syncope is believed to be excessive parasympathetic stimulation [2]. More specifically, the mechanism behind swallow syncope is suspected to be impulses conducted through reflex arcs between sensory fibers of the esophageal branches and cardiac branches of the vagus nerve leading to inappropriate parasympathetic vagal stimulation with subsequent suppression of cardiac heart rate and contractility [2]. Our patient's development of progressive bradycardia with anesthesia induction supports vagal hypersensitivity as an underlying factor in the pathogenesis of her swallow syncope and the presence of a hiatal hernia with gastric fundus herniating into the space located directly between the heart and esophageal and vagal plexus strongly aligns with this theory. However, no mention of a hiatal hernia was made in the aforementioned report of swallow syncope associated with laparoscopic sleeve gastrectomy [5]. An oropharyngeal mechanism responsible for swallow syncope has also been proposed. In one study, investigators used electrophysiological testing to measure the time between initiation of swallowing and onset of bradycardia or asystole, with results showing the delay to be approximately two to three seconds [8]. This relatively short delay in onset points toward the oropharynx as the location from which initial nerve signaling originates, although it is believed that the oropharyngeal and lower esophageal origination points are a continuum of the same esophageal nerve plexus. 

Decrease in BMI leading to metabolic feedback loops has also been suggested as the cause of sinus bradycardia following bariatric surgery [6]. Severity of bradyarrhythmia increased as amount of weight loss postoperatively increased [7,8]. One study found that patients who developed sinus bradycardia following bariatric procedure had an average decrease in BMI of 34.4% [7]. Studies did not distinguish swallow syncope from sinus bradycardia or other bradyarrythmias. In the case of our patient, pre-procedure weight of 135.9 kg decreased significantly to 122.7 kg at time of presentation, with weight loss totaling 13.2 kg (9.02%) over approximately six weeks. The pathophysiology responsible for the correlation between weight loss and severity of bradyarrhythmia is suspected to be changes in GLP-1 and leptin levels [9]. However, a recent meta-analysis of 27 trials including a total of 70,299 patients found no increased risk of atrial or ventricular arrythmias with GLP-1 agonist use [10]. A hereditary component is also possible, as evidenced in our case, as well as one other reported case of swallow syncope [11]. A twin study including 51 same-sex twin pairs showed a significant higher concordance in monozygotic twins than in dizygotic twins in syncope associated with typical vasovagal triggers (p=<0.001) [12].

Few treatment guidelines specific to reflex syncope, which includes the situational forms of syncope such as swallow syncope, have been published. The existing recommendations are based on evidence limited to case reports, case series, and small observational studies [1]. According to the 2017 American College of Cardiology (ACC)/American Heart Association (AHA)/Heart Rhythm Society (HRS) guidelines for the evaluation and management of patients with syncope, treatment of reflex syncope is centered on avoidance of triggers, increased salt and water intake, and reduction or removal of diuretics and antihypertensive medications [1]. Reversible underlying etiology should be corrected whenever possible [1]. Although irritation of the esophageal and vagal plexus due to hiatal hernia is suspected to be responsible for our patient's syncopal events, it was not identified as a correctable cause during the patient's hospital admission. Furthermore, laparoscopic fundoplication for the correction of hiatal hernias is reserved for patients with Type II, III, and IV (paraoesophageal) hernias [13]. Staging is based on barium swallow radiography and is necessary to determine treatment course [13] and fundoplication may not have been the recommended approach in our patient's case. The ACC/AHA/HRS guidelines recommend pacemaker placement for the treatment of recurrent vasovagal syncope with asystolic pauses in patients over the age of 40 (Class IIb evidence), but there are no recommendations made pertaining to pacemaker placement in neuroinhibitory reflex syncope [1]. However, the International Study on Syncope of Uncertain Etiology 3 (ISSUE-3), a randomized double-blinded placebo-controlled trial did find a 32% absolute risk reduction of recurrent syncope following dual chamber pacemaker placement in patients with severe neurally mediated asystolic syncope [14]. All forms of reflex syncope were included, excluding carotid sinus syndrome [14]. The 2018 European Society of Cardiology (ESC) Guidelines for the diagnosis and management of syncope reserves treatment of reflex syncope with cardiac pacing for older patients with the dominant cardioinhibition subtype as evidenced by lack of prodrome [15].

## Conclusions

Our case of swallow syncope secondary to a bariatric procedure is one of two such cases reported in medical literature. Aspects of our case such as the progressive development of a large hiatal hernia, family history of swallow syncope secondary to Roux-en-Y procedure in the patient's twin sister, and successful treatment with dual chamber pacemaker placement distinguish it from other reported cases of swallow syncope. Genetic predisposition is possibly involved in the development of our patient's symptoms. Although a hereditary component in syncope has been previously studied, review of the literature revealed a lack of studies pertaining to the development of hiatal herniation, indicating there is a need for retrospective studies investigating for any genetic factors in patients with hiatal hernias. The choice to treat our patient's reflex syncope with dual chamber pacemaker placement was based on the need for immediate resolution of symptoms due to the severe impact her symptoms had on quality of life, although timely identification of the hiatal hernia may have influenced the decision to proceed with cardiac pacing. Clinicians should consider structural anatomical abnormalities and gastrointestinal sources of pathogenesis during their evaluation as this may reveal a reversible cause of syncope. This is especially true for patients presenting with bradyarrythmias following bariatric procedures. Further investigation is warranted to help guide screening and diagnosis. 
